# Building SuperModels: emerging patient avatars for use in precision and systems medicine

**DOI:** 10.3389/fphys.2015.00318

**Published:** 2015-11-06

**Authors:** Sherry-Ann Brown

**Affiliations:** Division of Cardiovascular Diseases, Department of Medicine, Mayo ClinicRochester, MN, USA

**Keywords:** computational, avatars, virtual patient, precision medicine, systems medicine, supermodels, discipulus, electronic health record

## Introduction

**Figure 1:**
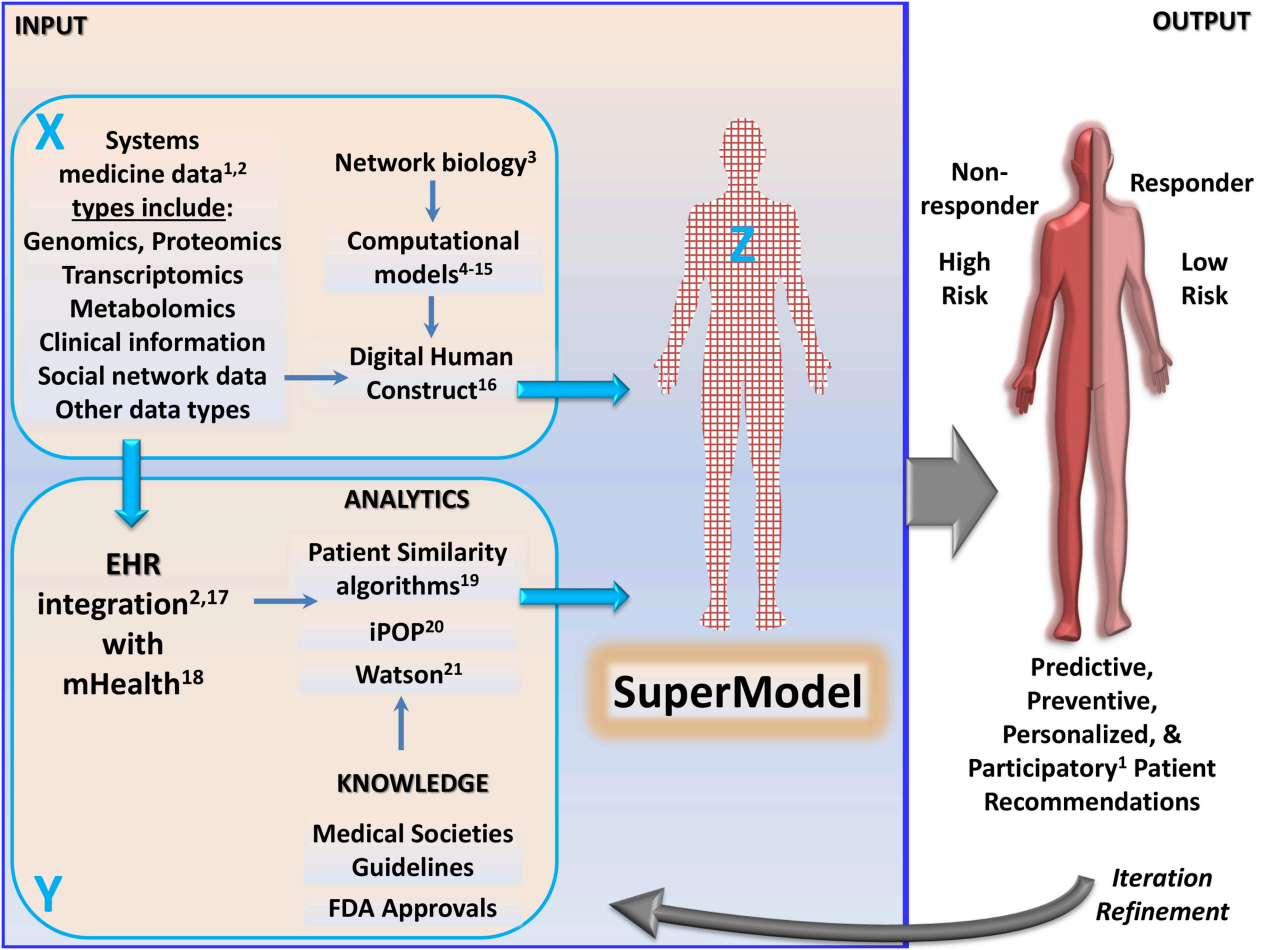
**Building SuperModels for precision and systems medicine, with incorporation of computational avatars**. *Input*: A feedforward loop (top left panel X connects to patient avatar Z both indirectly via bottom left panel Y and also directly, by large blue arrows) is fueled by two disparate paths. In the top left panel labeled X, various systems medicine data types, along with network biology and emerging computational models (including genome-scale metabolic models, Agren et al., 2014; Yizhak et al., 2015), provide input for patient representations such as the digital human construct being developed by The Discipulus Project. In the bottom left panel labeled Y, systems medicine data are integrated into patients' mobile health (mHealth) technologies and electronic health records (EHR) (Brown et al., 2015b). mHealth and EHR data are coupled with external knowledge [e.g., from medical societies guidelines and Food and Drug Administration (FDA) approvals] by cognitive machines such as Watson, and analytics are employed to process multi-omics (integrated personal omics profile; iPOP) and patient similarity algorithms. The path labeled Y is already in progress with EHR data, independent of digital human constructs described in the path labeled X. Paths X and Y can be bridged by locally supervised metric learning (LSML) similarity measures and similarity network fusions (SNF), for synergistic creation of SuperModels to produce results that cannot be obtained from path X or path Y alone. *Output*: High-yield predictive, preventive, and personalized data indicate patients at low/high risk for disease/adverse effect development. Individualized therapeutic plans can therefore be devised, also guided by the patient's likelihood of being a responder or non-responder to specific medications. Provision of personalized data should be in the context of systems medicine counseling, integrating genetic counseling with information about various forms of systems medicine data (Brown et al., 2015b). *Iteration*: The curved gray arrow linking output to input represents using outcome observations to iterate and refine SuperModels at all stages of development, to guide precision medicine. ^1^Hood and Flores (2012), ^2^Brown et al. (2015b), ^3^Barabási and Oltvai (2004), ^4^Schuyler et al. (2011), ^5^Plotkin et al. (2013), ^6^Bikson et al. (2012a), ^7^Brown and Loew (2014), ^8^Brown et al. (2015a), ^9^Henderson et al. (2014), ^10^Tortolina et al. (2012), ^11^Stamatakos et al. (2010), ^12^El-Kareh and Secomb (2000), ^13^Utsler (2015), ^14^Agren et al. (2014), ^15^Yizhak et al. (2015), ^16^The Discipulus Project (2013), ^17^Kullo et al. (2013), ^18^Steinhubl et al. (2015), ^19^Zhang et al. (2014), ^20^Chen et al. (2012), ^21^Savage (2014).

The era is approaching when each individual can be mapped to a patient avatar—not a life-sized 3D blue form of the patient filled with physical substance (as in the movie “Avatar”), but a hologram of the patient that simulates key medical components. Patient avatars will be composed of computational models combined with various data types and analytics to form what might be called SuperModels (Figure 1). These SuperModels (comprehensive virtual representations of the patient, not fashion models) will be important to help realize visionary precision medicine initiatives that have recently been announced (Collins and Varmus, 2015; Nature Biotechnology, 2015).

Precision medicine tailors prevention, diagnosis, therapeutics, and prognosis for each patient (Garay and Gray, 2012; Highnam and Mittelman, 2012; Mirnezami et al., 2012). Related to precision medicine is systems medicine (Auffray et al., 2009; Capobianco, 2012; Emmert-Streib and Dehmer, 2013; Wolkenhauer, 2013), which leverages systems biology (Noble, 2008) for clinical application, with resulting data termed “systems medicine data” (Brown et al., 2015b). Systems biology studies the behavior of organisms or cells as whole systems, and uses various advances in biotechnology, including genomics, transcriptomics, proteomics, metabolomics, methylomics, microbiomics, and elucidation of cellular interaction networks by network biology (Figure 1, top left panel labeled X). Often, systems medicine data from these various advances can be modeled and simulated with complementary computer science, mathematics, chemistry, physics, and engineering concepts in computational biology.

A variety of fields have used computational models as virtual surrogates of specific portions of patient physiology. These individual models can be considered computational avatars of a subset of the patient's organic identity. This is akin to cancer avatars in mice, which involve mouse models mapped to individual patients, for example, by injection of tumor cells from a particular individual. These cancer avatars facilitate personalized study of the pathophysiology and response to drugs of a particular patient's cancer cells. Similarly, biomathematical or computational cancer avatars simulate the micro-environment of individualized cancer cells.

Beyond such *in silico* exemplars, a computational avatar may also be thought of as any finite representation of a specific portion of the patient, that harnesses computing power. This includes electronic health records (EHR), patient portals, and a variety of other precision medicine tools. However, this paper focuses on individual biomathematical models as computational avatars that can be incorporated into comprehensive patient avatars for use in precision and systems medicine. The following section describes a non-exhaustive sample of biomathematical models, including genome-scale metabolic models (GEMs) that use computational approaches to integrate omics data (Yizhak et al., 2015). These computational avatars can serve as ingredients for SuperModels, forming the first portion (X → Z) of a positive feedback loop in Figure 1.

## Computational avatar exemplars

Several biomathematical models focus on understanding mechanism and prediction of pathophysiology progression, as well as delivery, efficacy, and adverse effects of therapeutics, such as deep brain stimulation or chemotherapy. Many of these computational models can replicate biomedical and/or electrophysiological properties of brain, cancer, and heart cells, personalized for each patient. Strategies and predictions for survival or for safer and more efficacious and well-timed therapy are studied and influence care of neurological and cardiovascular disorders and cancer, among others.

### Brain

Computational models of the brain have been developed, e.g., for amyotrophic lateral sclerosis (ALS). These models use known familial ALS mutations to predict functional implications or patient survival, based on mechanical properties of the mutant proteins that would be nearly impossible to produce experimentally (Schuyler et al., 2011; Plotkin et al., 2013). Thus, computational power is harnessed to predict survival and function in ALS.

Customized computational models of transcranial direct current stimulation (tDCS) have also been created (Bikson et al., 2012a,b). This non-invasive electrotherapy limits anatomic and temporal exposure to electricity in specific brain regions, minimizing side effects that could otherwise be experienced from pharmacotherapy (Bikson et al., 2012b). The tDCS computational avatars provide opportunities to individualize therapy for stroke, Parkinson's disease, and treatment-resistant depression, among others (Fregni et al., 2006; Truong et al., 2013; Tortella et al., 2015). Using these computational avatars to customize tDCS for patients at extremes of age or those with skull defects or brain damage could become standard tools to guide trials and therapy (Bikson et al., 2012a).

Another example of integrating modeling with experimental observations and clinical findings lies in spinocerebellar ataxia (SCA). The SCA modeling suite explains, interprets, or predicts experimental results in mouse models, post-mortem human brains, and peripheral blood samples from living patients (Brown and Loew, 2012, 2014). The suite is an example of the utility of computational systems biology in translational medicine (Brown et al., 2015a).

### Cancer

A number of computational avatars have also been designed for precision cancer care. These include models for colon cancer that synthesizesmathematical modeling, omics, other systems biology approaches, and pharmacologics to produce personalized molecular imprints aimed at predicting the right diagnostics and prescriptions (Tortolina et al., 2012; Henderson et al., 2014; American Cancer Society, 2015). With further study, these could be used to tailor therapy. The ContraCancrum project has moved in this direction with clinical trials illustrating the utility of computational models for lung carcinoma and other cancers (Stamatakos et al., 2010). Lung cancers account annually for ~15 and ~30% of all new cancer cases and deaths, respectively; colon cancer accounts for ~10% of all new cancer cases and deaths annually (American Cancer Society, 2015). Thus, computational avatars have tremendous potential for individualizing care of cancers that account for a great proportion of morbidity and mortality in adult patients.

Adverse drug effects (ADE) on the heart or other organs limit the administration of optimal pharmacologics for cancer care (Vejpongsa and Yeh, 2014). As an example to counteract this, mathematical models predict the optimal modes of doxorubicin delivery (El-Kareh and Secomb, 2000) for breast cancer, which annually accounts for 30 and 15% of all new cancer cases and deaths, respectively (American Cancer Society, 2015). Consistent with model predictions, liposomal delivery has subsequently been studied in a number of clinical trials, which have shown superior toxicity profiles compared to standard non-liposomal delivery for breast cancer (Lao et al., 2013). Computational avatars can therefore be used to predict and hopefully prevent ADE in cancer care.

Additional avatars may focus on pancreatic and hepatocellular cancer. Recent proteomic results implicated Glypican-1 as an unparalleled near perfect non-invasive diagnostic and screening tool detection of early pancreatic cancer (Melo et al., 2015). Addition of this and other biomarkers and systems medicine data to computational avatars will help guide safe, early, and effective cancer therapy. For example, personalized computational models based on proteomics data have predicted potential drugs to treat hepatocellular cancer, one of which has already been validated experimentally (Agren et al., 2014).

### Heart

Computational avatars have also been composed for the heart. The cardioid project from the International Business Machines (IBM) Corporation uses advanced computing to compose individualized 3D models of the heart (Utsler, 2015). The system is devised to predict the risk of sudden cardiac death due to Torsade de pointes or similar arrhythmic complications. These arrhythmias are another form of cardiotoxicity, in this case related to prolongation of the QT interval (distance between the start of the wave labeled “Q” and the end of the wave labeled “T” on an electrocardiogram), induced by drugs (e.g., antibiotics). Cardioid is thought to be the world's most detailed real-time human heart simulation (Lawrence Livermore National Security, 2015). Cardioid complements prior human heart models, and expands the capabilities of avatars to geometric point-of-care. Such an achievement resulted from computational, natural, and life sciences teamwork among computational biologists, physicists, and mathematicians.

These examples of computational avatars for the brain, heart, and cancer provide evidence for established units, which can 1 day be merged (e.g., with immersive virtual environment technology for 3D animated photorealistic virtual representations of the self, Fox et al., 2009) to form SuperModels for precision and systems medicine.

## Building supermodels

Computational avatars can be integrated with EHR or patient portals to build SuperModels, merging with clinical information about past medical history and diet and lifestyle habits, as well as measurements from wearable sensors, mobile health (mHealth) technologies (Steinhubl et al., 2015), and telemedicine (left section of X → Y in Figure 1). Some have termed a similar concept proposing patient mapping by integration of computational models with EHR information, and inviting incorporation of other biotechnological tools, as the “digital patient,” “virtual patient,” “medical avatar,” or “patient avatar” (The Discipulus Project, 2013). Digital patient platforms, similar to Discipulus (The Discipulus Project, 2013), will use 3D scanning to produce a virtual geometric and physiologic view of the patient. MRI and CT scan results will guide reproduction of individualized anatomy, organ structure, and temporal blood flow. This paper proposes that all of this information and all of these technologies can ultimately be amalgamated with knowledge sources (such as medical society guidelines documents) and analytics to create SuperModels as the most advanced patient avatars. This forms the second portion (X → Y → Z) of a positive feedforward loop in Figure 1.

Systems medicine EHR data can provide input for natural-language processors, such as Watson (Savage, 2014; Figure 1, bottom panel labeled Y). Cognitive machines like Watson assimilate patient information to tailor medical recommendations, guidelines, and treatment options to the individual (Savage, 2014). Watson is outfitted with virtual advisors trained by medical experts, to assist with personalized risk factor identification and associated recommendations. Cognitive machines and analytics are also employed to use machine learning and natural language processing in patient similarity algorithms that yield a cohort of patients similar to a target patient, stratified by medical conditions of most concern to the engaged target patient in participatory medicine. Integrative personal omics profile (iPOP) longitudinal analysis can also combine the various omics data integrated in the EHR to uncover extensive, dynamic changes over time across healthy and diseased conditions for the target patient (Chen and Snyder, 2013), and for similar patients.

Bridging the two paths (labeled X and Y in Figure 1) to create SuperModels can be achieved with implementation of methodologies such as locally supervised metric learning (LSML) similarity measures and similarity network fusions (SNF). LSML and SNF facilitate personalization and prediction for risk factor profiles and computational avatars by constructing networks of patient samples for a variety of available data types, and efficiently fusing data types into one representative network that captures the full pathophysiological spectrum, respectively, while harnessing the power of complementarity in the data (Wang et al., 2014; Ng et al., 2015). Both LSML and SNF substantially outperform single data type analysis, and models created from global datasets that do not address patient similarity, respectively, while establishing integrative pathways (Wang et al., 2014; Ng et al., 2015). Synergistically not additively combining EHR integration, knowledge sources, and analytics with systems medicine data, network biology, computational models, and digital human constructs in this way produce a novel modeling perspective that can be considered the advent of SuperModels. Emergent properties of such a powerful combination are the epitome of systems medicine.

SuperModels can be interrogated to determine whether an individual might be at low or high risk for developing serious side effects to certain medications, or whether a patient is likely to respond—or not respond—to chemotherapy, for example. SuperModels will therefore in part serve as a clinical decision support tool for shared decision-making, supporting patient engagement in participatory medicine. Participatory medicine, which advocates for patient input and education in all phases of their individualized care, is a component of P4 (predictive, preventive, personalized, and participatory) medicine, which has been proposed as the clinical face of Systems Medicine (Hood and Flores, 2012).

## Charting a course forward

Development of SuperModels will require worldwide partnerships in academia and industry, for creation, education (e.g., https://sems.uni-rostock.de/reproducible-and-citable-data-and-models/, implementation, and troubleshooting challenges. Accordingly, large interdisciplinary consultation meetings and online fora like those of the Discipulus project initiative will become the norm, bringing together clinicians, scientists, mathematicians, bioengineers, technologists, and patients (The Discipulus Project, 2013), and may ultimately engage crowd sourcing. Difficulties, such as assuring accuracy post-data-processing (Capobianco, 2012) involving (1) merger of multi-scale noisy biased data sets with small sample sizes and large amounts of measured data (Wang et al., 2014), (2) harmonization of whole-body pharmacokinetics and pharmacodynamics with cellular network and tissue-level models (Agren et al., 2014) and diverse systems medicine data types to form digital human constructs (Figure 1, top left panel labeled X), (3) robust cross-validation of highly complex model findings including temporal features of more diversified disease targets (Ng et al., 2015), and (4) intercalation with analytics (Figure 1, bottom left panel labeled Y), along with other systems medicine challenges (Capobianco, 2012) that may be encountered when building SuperModels (Figure 1, X → Z and Y → Z), will most effectively be addressed through collaborative efforts. These and other principles, including ones for efficiency and cost-effectiveness, will be needed to guide the use of SuperModels in systems medicine (see companion paper in Frontiers in Genetics, Brown, in review), along with ethical and other considerations for EHR integration (Kullo et al., 2013).

## Author contributions

SB conceived of, analyzed, designed, drafted, critically revised, approved, and agreed to be accountable for this submitted work.

### Conflict of interest statement

The author declares that the research was conducted in the absence of any commercial or financial relationships that could be construed as a potential conflict of interest.
